# locuszoomr: an R package for visualizing publication-ready regional gene locus plots

**DOI:** 10.1093/bioadv/vbaf006

**Published:** 2025-01-16

**Authors:** Myles J Lewis, Susan Wang

**Affiliations:** Centre for Experimental Medicine & Rheumatology, William Harvey Research Institute, Queen Mary University of London, London EC1M 6BQ, United Kingdom; Centre for Experimental Medicine & Rheumatology, William Harvey Research Institute, Queen Mary University of London, London EC1M 6BQ, United Kingdom

## Abstract

**Summary:**

Locuszoomr is an R package for visualizing and creating publication-ready regional gene locus plots similar to those produced by the original web interface ‘LocusZoom’, but running locally in R. Genetic or genomic data with gene annotation tracks are plotted via R base graphics or ‘ggplot2’, allowing flexibility and easy customization. Modular plotting functions enable layering of multiple GWAS plots such as for PheWAS, comparing GWAS with eQTL signals, and aligning multiple locus plots on the same page. Interactive plots can be visualized using ‘plotly’ enabling quick identification of data points for labelling. The ‘LDlink’ API (https://ldlink.nih.gov/) can be programmatically queried to obtain linkage disequilibrium (LD) data from the 1000 Genomes Project which is overlaid on plots. The package allows users to query Ensembl databases from Bioconductor or AnnotationHub for genomic information, so it can be used to show gene track information for any species available in Ensembl. Helper functions enable recombination rate data from UCSC to be overlaid on plots. The package has a detailed vignette and includes embedded example GWAS data to allow users to try out different features. Locuszoomr is also fast: identifying 50 peaks in a GWAS of around 8 million SNPs takes around 0.6 s. Exporting 50 locus plots with 1 Mb window with recombination rate takes around 30 son Intel ninth gen CPU or 14 son Apple ARM M3.

**Availability and implementation:**

The R package locuszoomr is open-source and available from CRAN: https://cran.r-project.org/package=locuszoomr and GitHub: https://github.com/myles-lewis/locuszoomr

## 1 Introduction

The locuszoomr package allows users to produce publication-ready gene locus plots similar to those produced by the popular web interface ‘locuszoom’ (Pruim *et al.* 2010), but running purely locally in R. While the original locuszoom team provided source code in R and python, installation of this code was not as simple as direct installation of a pre-made package in R and the original locuszoom codebase is no longer maintained. Many users continue to use locuszoom to generate regional locus plots via uploading subsets of genome-wide association study (GWAS) data to the locuszoom website (http://locuszoom.org). However, this is a legacy service which is no longer maintained and does not support the latest genome assemblies such as hg38. Locuszoomr was conceived to be easily installed as an R package from CRAN to allow any user to rapidly make use of it without having to link to python or other libraries. Furthermore, locuszoomr was written from the ground up to be able to plot gene track information from any Ensembl database, both older Ensembl databases included as standalone packages in distributions of Bioconductor and more recently distributed as downloadable SQL databases via AnnotationHub. While in recent years a javascript version locuszoom.js (Boughton *et al.* 2021) has been made available which is useful for making websites, most users do not have easy access to this version from within R. By writing locuszoomr as a pure R package, this allows users to quickly generate large numbers of regional genomic plots of GWAS or other genomic or transcriptomic data without having to upload subsets of data to the locuszoom web interface. It has also been designed specifically to allow stacking of plots and overlaying of multiple data types to enable comparison of multiple genome-wide data as genetics enters the era of phenome-wide association studies (PheWAS) and multi-omic studies. Thus, locuszoomr is a timely and essential advancement in the field.

## 2 Installation and required packages

Instructions for installation are provided in the README file which can viewed at the Github repository (https://github.com/myles-lewis/locuszoomr). It is important to install Bioconductor package manager first. Then the package can be installed from CRAN. Required and suggested R package dependencies are listed on the CRAN webpage for the locuszoomr (https://cran.r-project.org/package=locuszoomr).

### 2.1 Code for installation

if (! requireNamespace(“BiocManager”, quietly = TRUE))

   install.packages(“BiocManager”)

BiocManager::install(“ensembldb”)

BiocManager::install(“EnsDb.Hsapiens.v75”)

install.packages(“locuszoomr”)

## 3 Implementation

**Figure 1.**
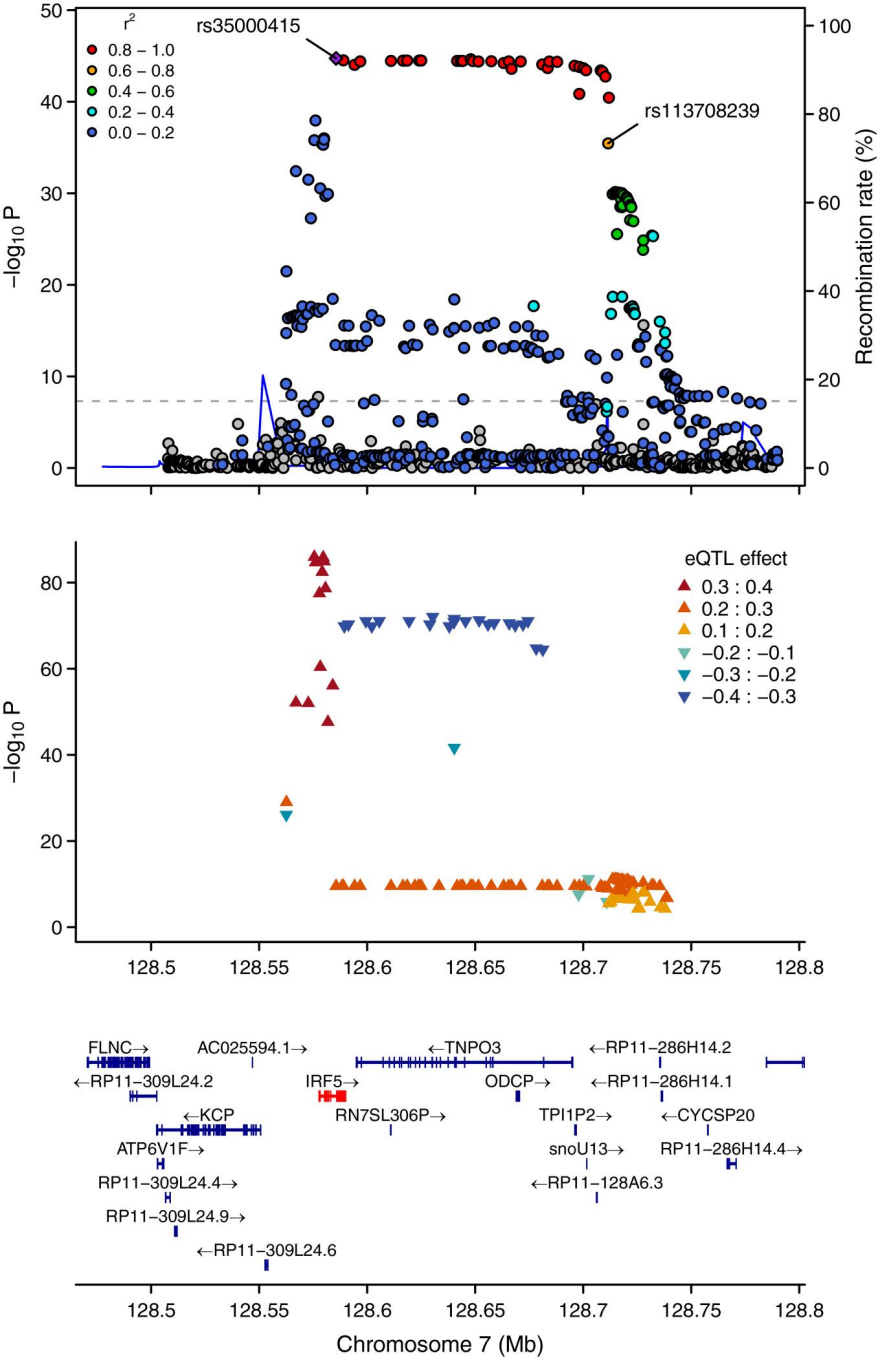
Example layered regional plot showing association between *IRF5* and systemic lupus erythematosus (SLE) with an associated eQTL. GWAS data (upper panel) is from systemic lupus erythematosus (SLE) GWAS (Bentham *et al.* 2015) using GRCh37 genome reference. Middle panel shows eQTL data for this locus obtained from GTEx via LDlinkR: eQTL for *IRF5* expression is shown for whole blood. Bottom tracks show gene-exon structure alignment based on Ensembl version 75 with putative susceptibility gene *IRF5*. Genes in the gene track can optionally be highlighted in colours. Here *IRF5* is highlighted in red. See the README on Github for the code used to generate this example plot. The upper panel data are embedded in the package and the locus object was generated using command locus(). Recombination rate data was added using command link_recomb(). LD linkage information was added using link_LD(). EQTL data wer obtained using link_eqtl(). Note that link_LD and link_eqtl require the user to sign up for a user account on the NIH LDlink website. The final layered plot was exported using functions set_layers(), scatter_plot(), eqtl_plot(), and genetracks().

locuszoomr produces regional gene annotation plots via either R base graphics or ‘ggplot2’ and plots can readily be customized, labelled and stacked. In Fig. 1, GWAS data showing association of *IRF5* with susceptibility to systemic lupus erythematosus (SLE) (upper panel) is compared against eQTL data from this locus (middle panel) obtained from GTEx (Bentham *et al.* 2015, GTEx Consortium 2017). The eQTL shown is for *IRF5* expression in whole blood and data was obtained programmatically via LDlinkR using an interface function in locuszoomr. The bottom tracks show gene-exon structure alignment based on Ensembl version 75 with putative susceptibility gene *IRF5* highlighted in red. Sample code to allow users to quickly generate Fig. 1 using in-built data stored within the package is given in the README on the Github site for the package. Note that the linkage disequilibrium (LD) overlay and eQTL plot require users to sign up and obtain an access token from the NIH LDlink website (see below).

A browser-based interactive ‘plotly’ version of locus plots can also be generated, which is helpful for identifying specific single nucleotide polymorphisms (SNPs) of interest (or other genomic features) for labelling or downstream analyses. Other packages have been developed for visualizing regional genomic information such as ggbio, Gviz, sushi (no longer maintained), Plotgardener and topr which are available in R (Yin *et al.* 2012, Hahne and Ivanek 2016, Kramer *et al.* 2022, Juliusdottir 2023) and GeneticsMakeie.jl for Julia (Kim *et al.* 2023). However, these packages can be unwieldy for refining and exporting regional association plots for publication, and aligning multiple layered scatter plots and plots from different loci on the same page is either not supported or difficult to code. Gviz is suited to displaying genomic track data in a visual manner similar to genome browsers such as UCSC, but less suited to regional plots of association data. For example, placing a gene track underneath a scatter plot for a region of GWAS data can be complicated to code using previous packages and perfect alignment of the gene track coordinates with the scatter plot coordinates is not implemented by default. Locuszoomr correctly aligns GWAS or other genomic data against gene tracks in both R base graphics, ggplot2 and plotly.

### 3.1 Extension to diverse genomes

The original locuszoom website is restricted to older specific human genomes ie GRCh37 (hg19) and before, so that the latest human reference assembly GRCh38 (hg38) as well as genomes from other species are not supported. Ensembl version 75, which has not been updated since 2014, was the last GRCh37 version released and is increasingly out of date, while Ensembl version 112 (GRCh38) is, at time of writing, the current latest human genome annotation available. locuszoomr enables access to gene track information from all available Ensembl genome databases including the latest human genome builds as well as access a wide range of other species. Based on the ensembldb format, users would also be able to create their own annotation database for novel organisms or genomes and use locuszoomr to annotate plots even if no genome for that species is currently available in Ensembl.

### 3.2 Modular approach

The package has been written with modular functions, so that users can layer multiple scatter plots correctly aligned above each, for example to display stacked multiple GWAS results at the same locus or PheWAS results, or to compare local GWAS result against expression quantitative trait loci (eQTL) results at a particular locus. This feature has been enabled for both base graphics and ggplot2. Another common issue for users trying to generate finalized figures for publications, is trying to layout and align multiple locus plots from different loci on the same page, again implemented for both base graphics and ggplot2. The modular approach also includes several useful helper functions that allow users to mix and match grid graphical objects (‘grobs’), for example generated by ggbio, with ggplot2 scatter plots and locuszoomr gene tracks. The function gg_addgenes() can add gene tracks to an existing ggplot2 scatter plot giving users full customization over the scatter plot including labelling and legends.

### 3.3 Adding linkage disequilibrium, recombination rate, and eQTL information

Linkage disequilibrium (LD) data from the 1000 Genomes Project (Sudmant *et al.* 2015) can easily be accessed using the LDlink API, made possible through the LDlinkR package (Myers *et al.* 2020). This requires users to sign up on the NIH LDlink website (https://ldlink.nih.gov/?tab=apiaccess) to obtain an API access token. LD information is overlaid using a customisable colour scheme on the scatter plot. Alternatively, users can provide their own LD data. Similarly, recombination rate data can be programmatically obtained from UCSC for individual loci through API requests which are made via the rtracklayer Bioconductor package. However, for users who are plotting multiple loci we recommend that users download the full recombination rate track for the relevant human genome (hg19 or hg38) from UCSC. Section 7 (“Add recombination rate”) of the detailed package vignette, which is hosted on CRAN (https://cran.r-project.org/web/packages/locuszoomr/vignettes/locuszoomr.html), gives instructions for users on how to obtain the recombination rate data files for hg19 and hg38 from UCSC along with example code on how to deploy these bigwig datatype files to rapidly generate large numbers of locus plots. The package can then make use of these data files stored locally, which is much faster than making repeated API requests and less prone to network time-outs.

### 3.4 Other useful features

- Individual scatter plot points can be labelled with text and label lines.
- Recombination rate data from UCSC can be overlaid with a secondary axis similar to the original locuszoom plots.
- GTEx consortium multi-tissue eQTL data (GTEx Consortium 2017) can be obtained from the LDlink API and overlaid on plots.
- An extremely fast quickpeak function has been added to scan GWAS datasets for peaks allowing to users to rapidly output locus plots for all peaks.
- Symbols, colour and size of scatter plot points in the GWAS data plot can be customized, e.g. to show different symbols for imputed versus typed SNPs; or to show up or down triangles for directionality of association or directionality of expression for eQTL data.
- Gene track colours can be customized and specific genes highlighted. Genes can be restricted to specific types such as protein coding.
- Interactive ‘plotly’ outputs allow users to rapidly identify key scatter points simply by hovering over them.

The vignette is detailed and includes step by step example code for generating different plots. Documentation of each function is comprehensive. Included with the package is a small subset of data from three loci, *UBE2L3*, *STAT4*, and *IRF5*, from a systemic lupus erythematosus (SLE) GWAS (Bentham *et al.* 2015), which is used to demonstrate workable examples and generate plots in the vignette.

### 3.5 Programmed for speed

Locuszoomr is also fast. Applied to the SLE GWAS data (Bentham *et al.* 2015) downloaded from EBI which contains 7 915 251 SNPs, the quickpeak function identifies 50 peaks with nominal *P* < 1 × 10^−6^ in around 0.6 s on an Intel core i9 CPU. Overall, identifying peaks and exporting the 50 locus plots at these regions with 1 Mb window and with recombination rate to a single PDF file takes around 30.1 s on an Intel core i9-9900K (using pre-downloaded recombination rate file and GRCh37 reference) and around 13.9 s on Apple silicon M3 Arm64 processor. Applied to summary statistics from a larger GWAS such as a rheumatoid arthritis (RA) GWAS containing 21 727 440 SNPs (Ishigaki *et al.* 2022), quickpeak identified 105 peaks at *P* < 5 × 10^−8^ in 2.87 s, and locuszoomr exported 105 locus plots with recombination rate to a single PDF file in 161.3 s on Intel core i9-9900K. On Apple silicon M3 the same task took 0.805 s to find 105 peaks and 85.2 s to export 105 plots to PDF.

Since API requests for adding LD, recombination rate and eQTL data are slow and a bottleneck to the process of interrogating multiple loci, API requests are cached via the memoize package, so that if users request the same LD data or eQTL data again, the cache is invoked. This reduces server requests and speeds up analyses for users.

Finally, locuszoomr owes a debt to the original locuszoom whose clean and professional design aesthetic remains ideal for publication quality plots (hence its longevity and continued popularity). The locuszoomr package maintains this clean aesthetic while simplifying installation and improving functionality, speed and ease of use for R users.

## 4 Conclusions

We have developed a tool which is easy to install and allows users to rapidly inspect GWAS peaks and to cross-reference genetic and omics data at the same loci across multiple datasets. The tool can be used with any Ensembl database available through Bioconductor or AnnotationHub giving access to the latest genomic gene track data on a wide variety of species. The plotly version embedded within the package enables local interactive use to speed up identification of data points. It could also be used for full interactive data website deployment via shiny.

## Data Availability

Code used in this article is available from CRAN (https://cran.r-project.org/package=locuszoomr) and GitHub from https://github.com/myles-lewis/locuszoomr. SLE GWAS full summary statistics can be downloaded from EBI from https://www.ebi.ac.uk/gwas/studies/GCST003156.
